# Characterization and Survival Benefit of Drug Approvals for Metastatic Prostate Cancer, 2004 to 2022

**DOI:** 10.1177/11795549241227413

**Published:** 2024-02-06

**Authors:** David J Benjamin, Arash Rezazadeh Kalebasty

**Affiliations:** 1 Hoag Family Cancer Institute, Newport Beach, CA, USA; 2 Division of Hematology/Oncology, Department of Medicine, University of California, Irvine Medical Center, Orange, CA, USA

The incidence of metastatic prostate cancer is rising in several countries including the United States.^1^ As such, there is an urgent need for the development of newer therapeutics in treating metastatic prostate cancer, which is considered incurable. Due to groundbreaking research by Dr Charles Huggins and Dr Clarence Hodges in the 1940s, the cornerstone of treating metastatic prostate cancer has been hormonal therapy through androgen deprivation therapy (ADT).^2^ Since 2004, several new agents have received approval by the US Food and Drug Administration (FDA) and European Medicines Agency (EMA) for treating metastatic castrate-sensitive cancer (mCSPC) and castrate-resistant prostate cancer (mCRPC) including chemotherapies and androgen receptor signaling inhibitors (ARSIs). Despite multiple drug approvals over the past 2 decades, however, it remains unclear how much improvement in survival these therapies have offered.

To further investigate the survival benefit of therapies approved between 2004 and 2022, we reviewed the FDA’s website of cancer drug approvals.^3^ We confirmed these approvals being currently accepted standard-of-care therapies for mCSPC and mCRPC by reviewing expert consensus recommendations published by the National Comprehensive Cancer Network (NCCN).^4^ We collected data on progression-free survival (PFS) and overall survival (OS) from the FDA approval notification or from the corresponding clinical trial’s publication cited for drug approval.

Between 2004 and 2022, there were 14 drug approvals for metastatic prostate cancer either a single therapy or in combination. As of 2023, the FDA has approved the following 3 ARSIs for the treatment of mCSPC: abiraterone, enzalutamide, and apalutamide based off data from the LATITUDE, ENZAMET, and TITAN trials, respectively.^5^ In addition, the FDA has approved the triplet therapy regimen darolutamide plus docetaxel plus ADT based off data from the ARASENS trial.^6^ The average PFS benefit for patients with mCSPC from these trials was 28.4 months. Although OS was not reached in either treatment arm of ARCHES with enzalutamide, the OS benefit of adding abiraterone to ADT was 16.8 months per LATITUDE. Of note, the comparator arm in the studies that evaluated the 3 ARSIs was ADT alone, whereas the comparator arm in the ARASENS trial was placebo plus docetaxel plus ADT. The PFS and OS benefits from each therapy approved in the mCSPC setting can be seen in Table 1.

**Table 1.** List of FDA approvals for treatment of metastatic prostate cancer, 2004 to 2022.

Drug name	Year of approval	Indication	Primary outcome	Control arm	PFS benefit (months)	OS benefit (months)
Docetaxel	2004	mCRPC	Overall survival	Mitoxantrone	Not reported	2.4
Cabazitaxel	2010	mCRPC	Overall survival	Mitoxantrone	1.4	2.4
Sipuleucel-T	2010	mCRPC	Overall survival	Placebo	Not reported	4.1
Abiraterone	2011	mCRPC after chemotherapy	Overall survival	Placebo	1.9	3.9
Abiraterone	2012	mCRPC without prior chemotherapy	Radiographic progression-free survival and overall survival	Placebo	2.1	Not reached vs 27.2 (HR = 0.75, 95% CI = 0.61-0.93, *P* = .01)
Abiraterone	2018	mCSPC	Overall survival	Placebo plus ADT	18.2	16.8
Enzalutamide	2012	mCRPC	Overall survival	Placebo	10.9 (ARCHES); 56 (ENZAMET)	Not reached in either group in ENZAMET or ARCHES (HR = 0.66, 95% CI = 0.53-0.81, *P* < .001)
Enzalutamide	2019	mCSPC	Overall survival	Non-steroidal antiandrogen therapy (standard care)	Not reached vs 19.4 (HR = 0.39, 95% CI = 0.3-0.5, *P* < .0001)	Not mature
Radium-223	2013	mCRPC	Overall survival	Placebo	Not reported	3.6
Apalutamide	2019	mCSPC	Radiographic progression-free survival and overall survival	Placebo plus ADT	Not reached versus 22.1 (HR = 0.48, 95% CI = 0.39-0.6, *P* < .0001)	Not reached (HR = 0.67, 95% CI = 0.51-0.89, *P* = .0053)
Olaparib	2020	HRR + mCRPC	Radiographic progression-free survival	Enzalutamide or abiraterone	3.8	4.4
Rucaparib	2020	BRCA + mCRPC	Objective response rate and PSA response	No control arm (TRITON2)	Not reported	Not reported
177-Lu-PSMA	2022	mCRPC	Radiographic progression-free survival and overall survival	Chemotherapy, immunotherapy, Radium-223, or investigational drugs	5.4	4
Darolutamide plus docetaxel	2022	mCSPC	Overall survival	Placebo plus docetaxel plus ADT	Not reported	Not reached vs 42.4 (HR = 0.68, 95% CI = 0.57-0.80, *P* < .0001)

Abbreviations: ADT, androgen deprivation therapy; CI, confidence interval; FDA, Food and Drug Administration; HR, hazards ratio; mCRPC, castrate-resistant prostate cancer; mCSPC, metastatic castrate-sensitive; OS, overall survival; PFS, progression-free survival.

In contrast, there were 10 therapy approvals for mCRPC from 2004 to 2022. Among these approvals were 2 chemotherapies (docetaxel and cabazitaxel), 2 poly ADP ribose polymerase (PARP) inhibitors (olaparib and rucaparib), 2 ARSIs (enzalutamide and abiraterone, with the latter receiving 2 separate approvals before or after chemotherapy), 1 radioisotope treatment (Radium-223), 1 radioligand treatment (177-Lu PSMA), and 1 autologous stem cell therapy (sipuleucel-T). The average PFS benefit from these therapeutic approvals was 3.7 months (range = 1.9-5.4) and the average OS benefit for mCRPC was 3.67 months. The PFS and OS benefits from each therapy approved in the mCRPC setting can be seen in Table 1.

Ours is the first study, to our knowledge, to evaluate the survival benefit of therapies approved from 2004 to 2022 in treating metastatic prostate cancer. As anticipated, the average PFS benefit seen in approved therapies for mCSPC was longer than the average PFS benefit for treatments approved for mCRPC likely owing due to significant differences in tumor biology between castrate-sensitive and castrate-resistant prostate cancer.^7^ There are, however, several limitations to the study. The first limitation is variation between the comparator arms for several of the trials that evaluated therapies for treating mCRPC. Moreover, some therapies such as rucaparib had no comparator arm. An additional limitation of the study was that several studies are currently ongoing, with the median PFS or median OS not yet reached. Finally, another study limitation was that several studies including docetaxel, rucaparib, radium-223, and sipuleucel-T for mCRPC as well as triplet therapy (darolutamide plus docetaxel plus ADT) for mCSPC did not report the median PFS found in their respective studies which led to approval.

As shown in Table 1, the primary end point of recent clinical trials published since 2019 has moved away from OS toward surrogate end points such as radiographic PFS. In fact, out of 8 trials that led to drug/therapy approvals between 2004 and 2018, only 1 drug (abiraterone for mCRPC without prior chemotherapy) had a primary endpoint other than OS alone. However, among the 6 drug/therapy approvals from 2019 to 2022, 4 (75%) had a primary endpoint other than OS alone. Although the PREVAIL study suggested that radiographic PFS is a meaningful endpoint in mCRPC, there still remains debate whether this is a meaningful endpoint in designing trials for metastatic prostate cancer, particularly in the castrate-sensitive setting.^8^

Although these prostate cancer treatment approvals particularly for mCRPC may provide physicians and patients with additional treatment options, it is unclear if these additional therapeutic choices come at the cost of delaying the onset of palliative care and comfort care services.^5^ Moreover, given the growing recognition of financial toxicity with genitourinary cancer treatments such as in bladder cancer, it is unclear if some of these approved therapies add to health care costs in excess of their potential benefits for survival or quality of life.^9^ However, appraisal of these factors is beyond the scope of this study.

Although there have been PFS and OS benefits with 14 approved therapies for metastatic prostate cancer since 2004, survival benefit has been relatively modest for mCRPC. Median PFS and OS benefits may increase as several ongoing clinical trials mature and eventually present final survival data. Nonetheless, despite the survival benefits gained from these approved therapies, continued drug development particularly for metastatic castrate-resistant prostate cancer is warranted.
